# Insomnia, Daytime Sleepiness and Cardio-Cerebrovascular Diseases in the Elderly: A 6-Year Prospective Study

**DOI:** 10.1371/journal.pone.0056048

**Published:** 2013-02-14

**Authors:** Isabelle Jaussent, Jean-Philippe Empana, Marie-Laure Ancelin, Alain Besset, Catherine Helmer, Christophe Tzourio, Karen Ritchie, Jean Bouyer, Yves Dauvilliers

**Affiliations:** 1 Inserm, U1061, Montpellier, France; 2 Université Montpellier 1, Montpellier, France; 3 Paris Cardiovascular Research Centre (PARCC), University Paris Descartes, Sorbonne Paris Cité, UMR-S970, Paris, France; 4 Inserm, U897, Bordeaux, France; 5 Université Victor Segalen Bordeaux 2, ISPED, Bordeaux, France; 6 Faculty of Medicine, Imperial College, London, United Kingdom; 7 Inserm, CESP Centre for research in Epidemiology and Population Health, U1018, Reproduction and Child Development, Villejuif, France; 8 Univ Paris Sud, UMR-S1018, Villejuif, France; 9 CHU Montpellier, Service de Neurologie, Unité des Troubles du Sommeil, Hôpital Gui-de-Chauliac, Montpellier, France; INRCA, Italy

## Abstract

**Objective:**

To examine 1) the associations between history of cardio-cerebrovascular diseases (CVD) and insomnia complaints and excessive daytime sleepiness (EDS), and 2) the relationships between sleep complaints and future CVD in persons over 65.

**Methods:**

CVD was assessed at baseline and during two, four, and six-year follow-up in 5494 non-demented subjects. Self-reported insomnia complaints (poor sleep quality, difficulty in initiating sleep, difficulty in maintening sleep, and early morning awakening), EDS and sleep medication use were evaluated at baseline. Logistic regression models and Cox proportional hazard models, with delayed entry and age of participants as the time scale, were adjusted for socio-demographic, lifestyle and clinical variables.

**Results:**

At baseline, 748 participants had a past-history of CVD. A past-history of CVD was associated with EDS (OR = 1.28 95%CI = [1.05–1.57]) and the number of insomnia complaints (OR = 1.26 95%CI = [1.03–1.55] for 1–2 insomnia complaints; OR = 1.32 95%CI = [1.03–1.71] for ≥3 complaints). In longitudinal analyses, neither the four components of insomnia nor the number of insomnia complaints were significantly associated with first or recurrent CVD events (n = 391 events). EDS was independently associated with future CVD events even after adjusting for prescribed sleep medication and past-history of CVD (HR = 1.35 95%CI = [1.06–1.71]).

**Conclusion:**

Our results suggest that the relationships between sleep complaints and CVD could be complex. Insomnia complaints are more likely a consequence of CVD, whereas EDS appears to be a determinant of CVD independently of past-history of CVD. EDS screening may thus constitute a means of detecting persons at high risk of CVD.

## Introduction

Cardio-cerebrovascular disease (CVD) remains the largest cause of morbidity and mortality among adults, worldwide. [1] Epidemiological studies have established smoking, alcohol use, high blood pressure, diabetes mellitus, hypercholesterolemia and obesity as the most important risk factors for both coronary heart disease (CHD) and stroke. Although sleep disturbances may contribute to the development of CVD, they have not been included in models of cardiovascular risk prediction. [2], [3], [4], [5] Insomnia and excessive daytime sleepiness (EDS) are frequent sleep complaints which increase with age. [5], [6], [7] However, few studies have examined prospectively the association between sleep disturbances and CVD in community-dwelling persons. [8], [9], [10], [11] A large prospective study of over 50,000 adults without history of CVD followed-up over 11 years reported a significant association between insomnia symptoms and occurrence of acute myocardial infarction, but they found no association in participants over 65. [10] Another study in elderly subjects free of CVD failed to show a significant association between insomnia and CHD particularly after adjustment for depressive symptoms over a 3-year follow-up. [12] EDS was shown to be associated with an increased risk of incident CVD in the elderly without a history of CVD. [8], [9], [11], [13] However, to our best knowledge no previous studies have examined these associations in subjects with a past-history of CVD, raising the question of whether insomnia and EDS precede or are a consequence of CVD.

This large prospective study aims to examine the independent relationships between insomnia complaints, EDS and CVD events in community-dwelling persons over 65. These relationships can be complex as sleep complaints may be risk factors for CVD but conversely some sleep complaints may be consecutive to CVD. [14] Within the present study we aimed to 1) examine retrospectively the associations between a past-history of CVD and insomnia complaints and EDS, and 2) study the associations between insomnia complaints and EDS, and future CVD events in subjects both with (recurrent CVD events) and without (first CVD events) a past-history of CVD.

## Materials and Methods

### Ethics Statement

The study protocol was approved by the ethical committee of the University Hospital of Kremlin-Bicêtre, and written informed consent was obtained from each participant.

### Study Population

Subjects were recruited as part of the Three-City Study, an ongoing multi-site longitudinal study involving three French cities, Bordeaux, Dijon and Montpellier. [15] Briefly, 9294 subjects over 65 were recruited from the electoral rolls between 1999 and 2001. The participants were administered standardized questionnaires and underwent clinical examinations at baseline, two, four and six-year follow-ups.

### CVD Outcomes

At baseline, participants have a face-to-face standardized clinical interview including history of coronary artery disease (e.g. angina pectoris or myocardial infarction) and stroke.

At each 2-year follow-up visit, participants were asked to report new severe medical events or hospitalization since the last interview. For all subjects reporting a possible CVD, all available clinical information was collected, including emergency medical services, hospital records, neuroimaging reports (for stroke), and interviews with the patient’s physician or family. Incident CHD events included hospitalized angina or myocardial infarction, and CHD death. Stroke was defined, in accordance with the criteria of the World Health Organization, as a new focal neurological deficit of sudden or rapid onset and of presumed vascular origin, lasting 24 hours or more, or leading to death. Confirmed stroke cases were further classified as ischemic, hemorrhagic, or unspecified.

### Sleep Complaints

Sleep complaints were assessed at baseline as part of the clinical interview, followed by the completion of a sleep questionnaire. [2] The participants self-rated as “never, rarely, frequently, or often” occurrence of 1) being excessively sleepy during the day (EDS), 2) having difficulties in initiating sleep (DIS), 3) having difficulties in maintaining sleep during the night (DMS), 4) having early morning awakenings (EMA) without being able to go back to sleep, 5) snoring loudly. Participants also rated their sleep quality (SQ) as good, average or poor.

In the analyses, the presence of EDS was defined as reporting frequently/often being excessively sleepy. Sleep quality was dichotomized as poor versus good/average and other insomnia complaints (DIS, DMS, EMA) as frequently/often versus never/rarely. The number of insomnia complaints was defined by the number of dichotomized insomnia complaints.

### Medication at Baseline

An inventory of all drugs (prescription and over-the-counter drugs including sleep medication as well as antidepressant drugs) taken more than once a week over the preceding month was recorded at baseline. Medical prescriptions and the medications themselves were checked by the interviewer thus minimizing exposure misclassification. Sleep medication was classified as prescribed medication; benzodiazepine (BZD) and BZD-like compounds (zolpidem, zopiclone), antihistaminic compounds, miscellaneous medications (including hypnotics from different pharmacological families such as neuroleptics and antidepressants), as well as homeopathic and non-prescription treatments.

### Other Clinical and Biological Variables at Baseline

The standardized interview included questions on socio-demographic characteristics and current health status, respiratory and thyroid disorders, and mobility defined as social restriction (confinement to bed, home or outings restricted to neighborhood). [16] A life-style questionnaire was used to assess smoking status, alcohol and caffeine intake. Case-level depressive symptoms were defined as a score above the 16-point cut-off on the Center for Epidemiological Studies–Depression Scale (CES-D) [17], or current antidepressant treatment. Global cognitive function was assessed by the Mini-Mental State Examination (MMSE). [18] Blood was collected following overnight fasting, and centralized standard measurements of lipids and glucose levels were performed by investigators blind with respect to baseline characteristics. Blood pressure (BP) was measured at baseline twice after at least 5 minutes of rest in a seated position. The presence of hypertension was defined by systolic BP≥160 mmHg or diastolic BP≥95 mmHg or current antihypertensive treatment. Diabetes was defined as fasting glucose level ≥7.0 mmol/l or treatment with antidiabetic agents. Hypercholesterolemia was defined as total cholesterol level ≥6.2 mmol/L or treatment with lipid lowering agents.

A B-mode ultrasound of the carotid arteries was offered to participants aged ≤85 years. Mean intima media thickness (IMT) and the presence of plaques were assessed in the common carotid arteries as previously described. [19].

### Statistical Analyses

Associations between subject characteristics, sleep complaints (type and number of insomnia complaints and EDS) were quantified with odds ratios (OR) and their 95% confidence intervals (CI). Study center, age, gender and socio-demographic, clinical and biological variables associated with sleep complaints in univariate analysis (with p<0.15) were included in logistic regression models (EDS) or multinomial regression models (number of insomnia complaints) to estimate adjusted OR for the relationships between past-history of CVD and sleep complaints. When appropriate, the interaction terms were tested using the Wald-χ2 test given by the logistic regression model.

Cox proportional hazard models with delayed entry and age of the participants as the time scale were used to estimate hazard ratios (HR) and their CI for the associations between sleep complaints and risk of future CVD events. In the case of multiple events during follow-up, the first event was considered in the survival analysis. Two multivariate models were successively performed: 1) adjustment for gender, and center and 2) further adjustment for variables associated with the outcome (at p<0.15). In secondary analyses, fatal and non-fatal CVD, as well as future CHD and stroke events were evaluated as separate end points. Significance level was set at p<0.05. Analyses were performed using SAS statistical software (version 9.2; SAS Inc, Cary, North Carolina).

## Results

### Subjects Characteristics

**Figure 1:**
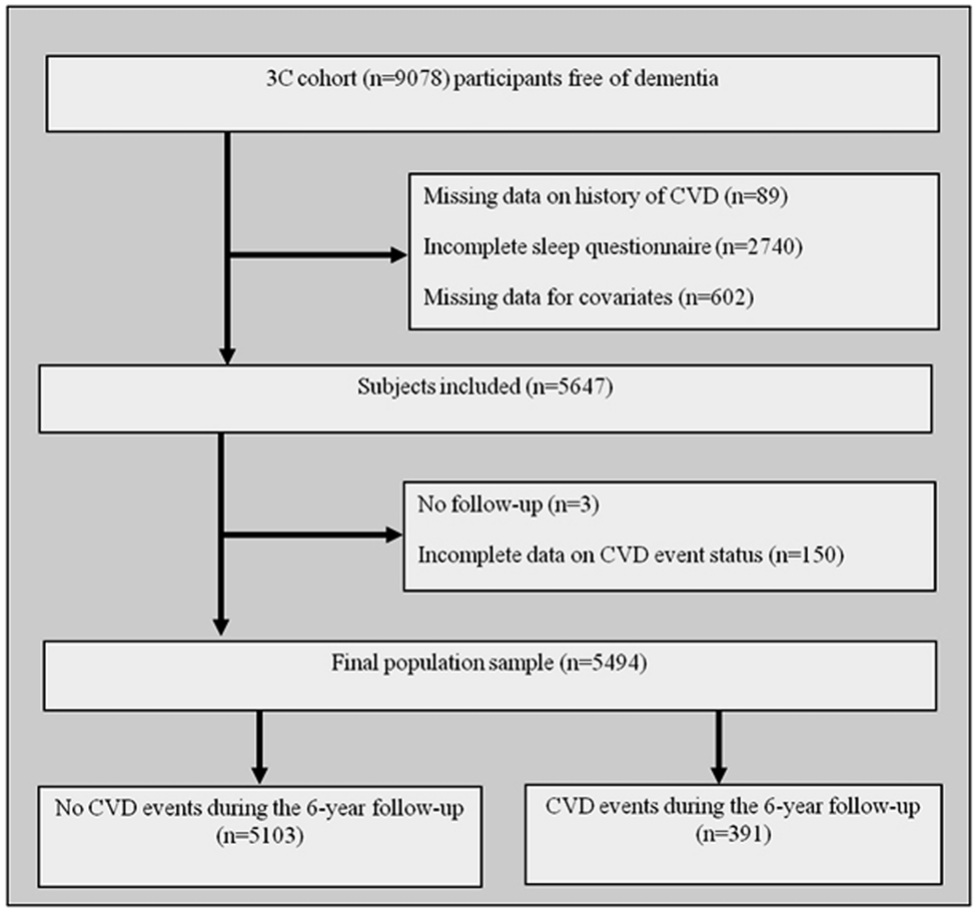
Flow diagram.

The study sample at baseline consisted of 5494 subjects with a median age of 72.8 years [range = 65.0–94.6] of whom 56.7% were women. These subjects were free of dementia, with information being available on CVD history, having fully completed the sleep questionnaire without missing data in adjustment covariates at baseline, and with at least one follow-up with information on a CVD event during follow-up (Figure 1). Subjects excluded from the study had a lower education level, were older, more frequently female, living alone, and with respiratory disease, hypertension, diabetes mellitus, depressive symptoms, cognitive impairment and mobility (p<0.0001 for all comparisons).

### Characteristics of Sleep Complaints

At baseline, 12.8% complained of poor SQ (of whom 99.9% reported another insomnia complaint), 34.4% had frequently/often DIS, 63.5% DMS, 35.9% EMA and 22.6% reported 3–4 insomnia complaints. EDS was reported by 18.1% of the subjects and 6.0% had both 3–4 insomnia complaints and EDS. Only, 16.5% of the participants used prescribed sleep medication at baseline (73.5% BZD, 23.9% BZD-like compounds, 8.8% antihistaminic compounds, and 12.2% miscellaneous medication). Among the subjects taking often sleep prescribed medication, 42.3% had 3–4 insomnia complaints, and 18.7% presented no insomnia complaints.

### Retrospective Analysis: Sleep Complaints and Past-history of CVD

Overall, 748 (13.6%) subjects reported a past-history of CVD at baseline including 547 with past-CHD, 156 with past-stroke and 45 with both past-CHD and past-stroke.

Baseline sociodemographic and clinical characteristics of the participants according to sleep symptoms (EDS and number of insomnia complaints) are described in Table 1. Subjects with sleep complaints were more likely to be confined to home, depressed, have chronic disorders (e.g. diabetes mellitus, respiratory, thyroid diseases), take prescribed sleep medication, snore loudly and consume less alcohol and caffeine (p<0.05 for all comparisons). Some characteristics were associated with EDS only, such as not living alone or obesity (p<0.02 for all comparisons) whereas others were only associated with the number of insomnia complaints such as low level of education, hypertension and hypercholesterolemia (p<0.04 for all comparisons). Subsequent analyses were thus adjusted for these factors plus others characteristics associated with EDS or number of insomnia complaints with p-value<0.15. Cognitive impairment as assessed with MMSE score was not significantly associated with EDS or the number of insomnia complaints.

**Table 1:** Baseline sociodemographic and clinical characteristics of participants according to sleep symptoms (Excessive daytime sleepiness (EDS) and number of insomnia complaints) evaluated at baseline.

	EDS						Number of insomnia complaints						Comparisons		
	No		Yes				0		1–2		3–4		1–2 vs 0	3–4 vs 0	
	N = 4500		N = 994				N = 1479		N = 2773		N = 1242				
Variable	n	%	n	%	OR [95%CI] (a)	p	n	%	n	%	n	%	OR [95%CI] (a)	OR [95%CI] (a)	global p
High level of education (b)	942	20.93	215	21.63	0.98 [0.82;1.17]	0.84	310	20.96	641	23.12	206	16.59	1.14 [0.97;1.33]	0.92 [0.75;1.13]	0.04
Living not alone ^(c)^	1333	29.62	293	29.48	1.22 [1.03;1.45]	0.02	395	26.71	770	27.77	461	37.12	1.02 [0.88;1.19]	1.18 [0.98;1.41]	0.14
Confined at home ^(c)^	178	3.96	75	7.55	2.01 [1.49;2.72]	<0.0001	41	2.77	129	4.65	83	6.68	1.42 [0.99;2.05]	1.81 [1.21;2.71]	0.01
Alcohol (g/day)															
≤12	2849	63.31	607	61.07	1	0.01	906	61.26	1652	59.57	898	72.30	1	1	0.02
12–36	1240	27.56	282	28.37	0.80 [0.67;0.96]		410	27.72	841	30.33	271	21.82	1.07 [0.92;1.25]	0.84 [0.69;1.03]	
>36	411	9.13	105	10.56	0.72 [0.56;0.94]		163	11.02	280	10.10	73	5.88	0.86 [0.69;1.09]	0.67 [0.48;0.92]	
Caffeine intake (mg/day)															
≤125	1137	25.27	303	30.48	1	0.03	357	24.14	712	25.68	371	29.87	1	1	0.01
125–375	2639	58.64	552	55.53	0.81 [0.68;0.95]		882	59.63	1622	58.49	687	55.31	0.95 [0.81;1.10]	0.74 [0.62;0.89]	
>375	724	16.09	139	13.98	0.84 [0.66;1.06]		240	16.23	439	15.83	184	14.81	0.99 [0.81;1.22]	0.77 [0.60;0.99]	
Smoker status ^(d)^	1791	39.80	468	47.08	1.02 [0.86;1.21]	0.81	625	42.26	1247	44.97	387	31.16	1.12 [0.96;1.30]	0.96 [0.79;1.16]	0.11
Respiratory disease ^(c)^	218	4.84	82	8.25	1.77 [1.34;2.34]	<0.0001	64	4.33	154	5.55	82	6.60	1.29 [0.95;1.74]	1.70 [1.20;2.41]	0.01
Thyroid disease ^(c)^	371	8.24	92	9.26	1.50 [1.16;1.94]	0.002	109	7.37	210	7.57	144	11.59	1.10 [0.86;1.41]	1.38 [1.05;1.82]	0.05
Depressive symptoms ^(c)^	1005	22.33	317	31.89	2.46 [2.08;2.92]	<0.0001	225	15.21	632	22.79	465	37.44	1.77 [1.49;2.10]	3.74 [3.08;4.54]	<0.0001
MMSE Score <26 ^(e)^	624	13.87	148	14.89	1.15 [0.94;1.41]	0.19	220	14.87	364	13.13	188	15.14	0.84 [0.70;1.01]	0.95 [0.76;1.18]	0.15
Body mass index															
Normal (<25)	2254	50.09	372	37.42	1	<0.0001	725	49.02	1292	46.59	609	49.03	1	1	0.06
Overweight (25–29)	1740	38.67	442	44.47	1.26 [1.07;1.48]		585	39.55	1106	39.88	491	39.53	1.02 [0.89;1.17]	1.08 [0.91;1.28]	
Obese (≥30)	506	11.24	180	18.11	1.91 [1.54;2.37]		169	11.43	375	13.52	142	11.43	1.22 [0.99;1.50]	0.94 [0.73;1.22]	
Hypertension ^(c)^	2610	58.00	644	64.79	1.13 [0.97;1.31]	0.13	815	55.10	1652	59.57	787	63.37	1.11 [0.97;1.26]	1.32 [1.12;1.56]	0.004
Diabetes mellitus ^(c)^	364	8.09	126	12.68	1.52 [1.21;1.91]	0.0003	115	7.78	279	10.06	96	7.73	1.31 [1.04;1.65]	1.14 [0.85;1.53]	0.06
Hypercholesterolemia ^(c)^	1691	37.58	365	36.72	1.00 [0.86;1.17]	0.96	541	36.58	996	35.92	519	41.79	1.00 [0.87;1.14]	1.18 [1.01;1.39]	0.04
Snoring loudly ^(f)^	1461	32.47	531	53.42	1.86 [1.60;2.16]	<0.0001	449	30.36	1101	39.70	442	35.59	1.41 [1.23;1.62]	1.13 [0.95;1.35]	<0.0001
Sleep medication ^(c)^	711	15.80	197	19.82	1.40 [1.16;1.68]	0.0005	137	9.26	387	13.96	384	30.92	1.55 [1.26;1.91]	3.83 [3.07;4.77]	<0.0001
EDS ^(f)^							114	7.71	550	19.83	330	26.57	2.53 [2.04;3.15]	3.65 [2.86;4.65]	<0.0001
Insomnia complaints															
0	1365	30.33	114	11.47	1	<0.0001									
1–2	2223	49.40	550	55.33	2.55 [2.05;3.17]										
3–4	912	20.27	330	33.20	3.64 [2.86;4.65]										

(a) Adjustment for age, center and gender.

(b) high vs low ^(c)^ yes vs no ^(d)^ past/current vs never ^(e)^ <26 vs ≥26 ^(f)^ frequently/often vs never/rarely.

Table 2 shows the associations between past-history of CVD and sleep complaints (number and type of insomnia complaints, EDS) evaluated at baseline. A past-history of CVD events was significantly associated with the number of insomnia complaints and DMS after multiple adjustments (model 3) and remained significant even after adjustment for EDS (model 4). In the same way, a past-history of CVD events was also associated with EDS in the complete model adjusted for the confounders including the number of insomnia complaints (model 4). No significant relationship was found between a past-history of CVD and the risk of having a “potential” obstructive sleep apnea syndrome (OSAS) defined clinically as being obese with frequent/often EDS and frequent/often loud snoring (n = 127) (OR = 1.34 95%CI = [0.76–2.36], p = 0.32).

**Table 2:** Relationships between past-history of CVD (stroke or CHD) and sleep complaints evaluated at baseline.

	Past-history of CVD, yes		Model 1	Model 2	Model 3	Model 4
Variable	n	%	OR [95% CI](a)	OR [95% CI](a)	OR [95% CI](a)	OR [95% CI](a)
Number of insomnia complaints						
0 (N = 1479)	157	10.62	1	1	1	1
1–2 (N = 2773)	405	14.61	1.37 [1.12;1.67]	1.29 [1.05;1.58]	1.26 [1.03;1.55]	1.24 [1.01;1.55]
3–4 (N = 1242)	186	14.98	1.71 [1.35;2.18]	1.43 [1.12;1.84]	1.32 [1.03;1.71]	1.28 [1.00;1.67]
Sleep quality (SQ)						
Good (N = 2654)	333	12.55	1	1	1	1
Average (N = 2136)	298	13.95	1.23 [1.04;1.47]	1.17 [0.98;1.40]	1.13 [0.95;1.35]	1.11 [0.94;1.34]
Poor (N = 704)	117	16.62	1.62 [1.28;2.05]	1.38 [1.07;1.77]	1.25 [0.97;1.62]	1.21 [0.93;1.56]
Difficulty with initiating sleep (DIS)						
Never/Rarely (N = 3603)	474	13.16	1	1	1	1
Frequently/Often (N = 1891)	274	14.49	1.39 [1.16;1.65]	1.23 [1.03;1.48]	1.15 [0.95;1.38]	1.14 [0.94;1.37]
Difficulty in maintaining sleep (DMS)						
Never/Rarely (N = 2003)	220	10.98	1	1	1	1
Frequently/Often (N = 3491)	528	15.12	1.38 [1.16;1.64]	1.26 [1.06;1.50]	1.25 [1.05;1.49]	1.23 [1.03;1.47]
Early morning awakening (EMA)						
Never/Rarely (N = 3522)	469	13.32	1	1	1	1
Frequently/Often (N = 1972)	279	14.15	1.15 [0.97;1.36]	1.05 [0.88;1.25]	1.00 [0.84;1.20]	0.99 [0.83;1.18]
Excessive daytime sleepiness (EDS)						
Never/Rarely (N = 4500)	558	12.40	1	1	1	1
Frequently/Often (N = 994)	190	19.11	1.47 [1.21;1.78]	1.29 [1.05;1.57]	1.28 [1.05;1.57]	1.25 [1.02;1.53]

(a) odds ratio (OR) for the presence of the sleep complaint indicated. The OR gives the risk of presenting a sleep complaint among those with a past-history of CVD compared to those without a past-history of CVD. The reference (OR = 1) corresponds to the subjects without the sleep complaint indicated.

Model 1 was adjusted for center, age and gender.

Model 2: Model 1 adjusted for educational level, living alone, mobility, alcohol intake, caffeine intake, smoking status, respiratory disease, thyroid disease, depressive symptoms, BMI, hypertension, diabetes mellitus, hypercholesterolemia, loudly snoring.

Model 3: Model 2 adjusted for prescribed sleep medication.

Model 4: Number of insomnia complaints, DIS, DMS and EMA were adjusted for variables of Model 3 plus EDS. EDS was adjusted for the Model 3 plus the number of insomnia complaints.

### Prospective Analysis: Sleep Complaints and Future CVD

Over the 6-year follow-up, 391 (7.1%) future CVD events were observed; 113 cases of stroke (28.9%) (89 ischemic, 23 hemorrhagic and 1 unspecified) and 278 of CHD (71.1%), including 14.8% fatal events. A total of 126 patients with a past-history of CVD (16.8%) had a recurrent event (29 stroke and 97 CHD) and 265 patients without a history of CVD (5.6%) had a first CVD event (84 stroke and 181 CHD).

Baseline socio-demographic and clinical characteristics of the participants according to the occurrence of CVD events during the follow-up are given in table 3.The risk of future CVD events increased significantly in subjects being obese, those confined to home, with respiratory disease, depressive symptoms, hypertension, diabetes mellitus, frequently using sleep medication, and with a past-history of CVD. Subsequent analyses were thus adjusted for these factors.

**Table 3:** Baseline predictors of CVD (CHD or Stroke) events during the follow-up.

	No future CVD		Future CVD			
	N = 5103		N = 391			
Variable	n	%	n	%	HR [95%CI] (a)	p
High level of education						
No	4033	79.03	304	77.75	1	0.16
Yes	1070	20.97	87	22.25	0.84 [0.66;1.07]	
Living not alone						
No	3570	69.96	298	76.21	1	0.86
Yes	1533	30.04	93	23.79	0.98 [0.76;1.26]	
Mobility						
Not confined	4882	95.67	359	91.82	1	0.0001
Confined	221	4.33	32	8.18	2.07 [1.42;3.02]	
Alcohol (g/day)						
≤12	3252	63.73	204	52.17	1	0.89
12–36	1390	27.24	132	33.76	1.02 [0.81;1.29]	
>36	461	9.03	55	14.07	1.08 [0.79;1.49]	
Caffeine intake (mg/day)						
≤125	1317	25.81	123	31.46	1	0.15
125–375	2978	58.36	213	54.48	0.80 [0.64;1.00]	
>375	808	15.83	55	14.07	0.87 [0.63;1.20]	
Smoking status						
Never	3054	59.85	181	46.29	1	0.63
Past/Current	2049	40.15	210	53.71	1.06 [0.84;1.33]	
Respiratory disease						
No	4837	94.79	357	91.30	1	0.01
Yes	266	5.21	34	8.70	1.58 [1.11;2.24]	
Thyroid disease						
No	4668	91.48	363	92.84	1	0.24
Yes	435	8.52	28	7.16	1.27 [0.85;1.87]	
Depressive symptoms						
No	3883	76.09	289	73.91	1	0.004
Yes	1220	23.91	102	26.09	1.41 [1.12;1.78]	
MMSE Score						
≥26	4385	85.93	337	86.19	1	0.68
<26	718	14.07	54	13.81	1.06 [0.80;1.42]	
Body mass index (kg/m^2^)						
Normal (<25)	2473	48.46	153	39.13	1	0.07
Overweight (25–29)	1996	39.11	186	47.57	1.26 [1.02;1.57]	
Obese (≥30)	634	12.42	52	13.30	1.31 [0.95;1.80]	
Hypertension						
No	2139	41.92	101	25.83	1	0.0001
Yes	2964	58.08	290	74.17	1.80 [1.43;2.27]	
Diabetes mellitus						
No	4690	91.91	314	80.31	1	0.0001
Yes	413	8.09	77	19.69	2.36 [1.84;3.04]	
Hypercholesterolemia						
No	3188	62.47	250	63.94	1	0.51
Yes	1915	37.53	141	36.06	1.07 [0.87;1.32]	
Snoring loudly						
Never/Rarely	3252	63.73	250	63.94	1	0.24
Frequently/Often	1851	36.27	141	36.06	0.88 [0.71;1.09]	
Prescribed sleep medication						
No	4274	83.75	312	79.80	1	0.0008
Yes	829	16.25	79	20.20	1.54 [1.19;1.98]	
History of stroke or CHD						
No	4481	87.81	265	67.77	1	0.0001
Yes	622	12.19	126	32.23	2.69 [2.17;3.34]	

(a) Adjustment for center and gender.

Table 4 shows the adjusted associations between sleep complaints at baseline and future CVD events over the 6-year follow-up. A significant positive association was observed between EDS and future CVD events (first and recurrent) even after adjusting for prescribed sleep medication and past-history of CVD (model 3). EDS was associated both with first CVD event in the sub-sample without CVD history at baseline (model 2, HR = 1.36 95%CI = [1.01–1.82], p = 0.04), and with recurrent CVD events among subjects with a past-history of CVD (n = 748) (model 2, HR = 1.47 95%CI = [1.01–2.18]). Associations between EDS, future CHD or stroke evaluated separately were significant after adjustment in model 2 (HR = 1.36 95%CI = [1.02–1.80], HR = 1.52 95%CI = [1.00–2.34], respectively) and borderline significant after adjustment in model 3 (HR = 1.30 95%CI = [0.98–1.73] p = 0.07, HR = 1.48 95%CI = [0.96–2.28] p = 0.08, respectively). EDS was associated with future non-fatal CVD (HR = 1.32 95%CI = [1.02–1.72]) but not with fatal CVD events (HR = 1.51 95%CI = [0.82–2.76], p = 0.18) after multivariate adjustment (model 3). In contrast to EDS, the number of insomnia complaints, SQ, DIS and DMS were not significantly associated with either future CVD events as a whole or with fatal or non-fatal CVD events after adjustment in model 3.

**Table 4:** Associations between sleep complaints and future CVD (CHD or Stroke) events over 6-year follow-up.

	No future CVD		Future CVD									
	N = 5103		N = 391		Model 0		Model 1		Model 2		Model 3	
	n	%	n	%	HR [95%CI]	p	HR [95%CI]	p	HR [95%CI]	p	HR [95%CI]	p
Number of IS(a)												
0	1381	27.06	98	25.06	1	0.53	1	0.93	1	0.98	1	0.88
1–2	2563	50.23	210	53.71	1.06 [0.83;1.35]		0.99 [0.77;1.26]		0.98 [0.76;1.25]		0.94 [0.74;1.20]	
3–4	1159	22.71	83	21.23	1.19 [0.88;1.61]		1.04 [0.76;1.42]		0.99 [0.72;1.35]		0.94 [0.69;1.29]	
DIS (b)												
Never/Rarely	3336	65.37	267	68.29	1	0.26	1	0.77	1	0.94	1	0.78
Frequently/Often	1767	34.63	124	31.71	1.14 [0.91;1.43]		1.04 [0.82;1.31]		0.99 [0.78;1.25]		0.97 [0.76;1.23]	
DMS (c)												
Never/Rarely	1879	36.82	124	31.71	1	0.15	1	0.46	1	0.48	1	0.72
Frequently/Often	3224	63.18	267	68.29	1.17 [0.95;1.46]		1.09 [0.87;1.35]		1.08 [0.87;1.34]		1.04 [0.84;1.29]	
EMA (d)												
Never/Rarely	3264	63.96	258	65.98	1	0.99	1	0.51	1	0.37	1	0.36
Frequently/Often	1839	36.04	133	34.02	1.00 [0.81;1.24]		0.93 [0.75;1.15]		0.91 [0.73;1.13]		0.90 [0.73;1.12]	
EDS (e)												
Never/Rarely	4219	82.68	281	71.87	1	0.0002	1	0.005	1	0.005	1	0.01
Frequently/Often	884	17.32	110	28.13	1.56 [1.23;1.97]		1.41 [1.11;1.79]		1.40 [1.11;1.78]		1.35 [1.06;1.71]	

Model 0 was adjusted for center and gender.

Model 1 was adjusted for model 0 plus mobility, respiratory disease, depressive symptoms, Body Mass Index, hypertension, diabetes mellitus.

Model 2 was adjusted for model 1 plus prescribed sleep medication.

Model 3 was adjusted for model 2 plus past-history of cerebro-cardio vascular disease (stroke or CHD).

(a) number of insomnia symptoms.

(b) Difficulty with initiating sleep.

(c) Difficulty in maintaining sleep.

(d) Early morning awakening.

(e) Excessive daytime sleepiness.

### Sensitivity Analyses

To assess whether hypertension could mediate the association between EDS and future CVD events, we performed stratified analysis by baseline hypertension status. However, EDS remained borderline associated with future CVD events (p = 0.06) in both subjects with (n = 3524) and without (n = 2240) hypertension, and interaction between hypertension, EDS and future CVD events was not significant (p = 0.60).

Elderly subjects clinically defined as being potentially “at risk of OSAS” (n = 127) were not associated with increased risk of future CVD events. Carotid IMT and plaques were measured in a sub-sample of 4 240 subjects under 85 years. The presence of carotid plaques at baseline were associated with future CVD events (HR = 1.97 95%CI = [1.55–2.52]) whereas carotid IMT was not (p = 0.37). In multivariate models further adjusted for the presence of carotid plaques, the association between EDS and future CVD events was borderline significant (HR = 1.29%CI = [0.98–1.69]).

## Discussion

In this study of community-dwelling older adults, retrospective analysis firstly showed an association between a past-history of CVD and EDS and insomnia complaints. Furthermore, prospective analyses indicated that EDS at baseline increased the risk of future CVD events after a 6-year follow-up by 1.4-fold, independently of confounding factors including past-history of CVD. In contrast, number and type of insomnia complaints were not associated with future CVD events. Our findings suggest that in the elderly the relationship between sleep disorder and CVD may be symptom-specific, with insomnia being a likely consequence of CVD and EDS *per se* predisposing elderly persons to future CVD.

Our retrospective analyses suggest that a past-history of CVD increases the risk of EDS and DMS by 25%. These associations are independent of socio-demographic, lifestyle characteristics, sleep medication and numerous other potential determinants of CVD. These results are in agreement with the high prevalence of sleep complaints, especially insomnia observed in persons with CVD. [3] They also confirm previous studies showing a higher prevalence of EDS in individuals with CVD [4] as well as DMS in stroke survivors. [14] In the latter study, stroke survivors also had a higher rate of non-restorative sleep and DIS, however, the analysis did not adjust for sleep medication. [14] In the current study, an association was observed between past-CVD and DIS in model 2 (p = 0.02) but not when further adjusting for sleep medication.

The number of insomnia complaints, SQ, DIS, EMA and DMS, were not associated with either first or recurrent CVD events. Our study confirmed the results of a smaller longitudinal study of 2960 persons over 65 with a shorter 3-year follow-up in which insomnia complaints (DIS, EMA, restless sleep) were not related to a first myocardial infarction. [12] In a larger study with a 11-year follow-up, frequent insomnia symptoms (DIS, DMS and feelings of non-restorative sleep) were significant predictors of a first acute myocardial infarction in adults. [10] However, these associations failed to reach significance in persons over 65, except for DIS, but the analyses were neither adjusted for sleep medication nor depressive symptoms. Our study extends these observations by showing that insomnia complaints in the elderly do not predict CVD whether stroke or CHD, fatal or nonfatal, first or recurrent. Thus it suggests that insomnia is more likely to be a consequence rather than a determinant of CVD.

EDS at baseline was associated with a 35% increased risk of future fatal and non-fatal CVD events independently of potential confounders, including sleep medication and past-CVD history. Our findings extend previous observations on first onset of CVD [8], [9], [11] reporting that EDS was a risk factor for future CVD events whatever the number of life-time CVD episodes. Hence, our results suggest that EDS *per se* is an independent risk factor of CVD and may thus constitute an early warning sign for future CVD.

The mechanisms linking EDS to future CVD events remained unclear as EDS may be due to a number of underlying causes e.g. obesity, depression, cognitive impairment, and chronic diseases and CVD. [20], [21] EDS is also frequently associated with sleep-disordered breathing (SDB). A large community-based cohort of adults over 40 showed that severe OSAS increased the risk of both fatal and nonfatal CVD over 10 years of follow-up [22] but this result was not observed in a population of subjects aged over 70. [23] Another longitudinal study in elderly reported an increased risk of all-cause mortality in subjects with both SDB and EDS but without association for subjects with SDB without EDS, and EDS without SDB. [24] In the absence of polysomnography assessment, we clinically defined persons potentially “at risk of OSAS” based on obesity, frequent EDS and loud snoring. We failed to observe an association with future CVD events although it should be acknowledged that obesity and loud snoring constitute less powerful predictors of OSAS in elderly. [25] The mechanisms that link EDS to CVD events may involve an activation of the hypothalamic-pituitary-adrenal axis and the sympathetic nervous system with an increased level of circulating catecholamines. Potential sympathetic over-activity during sleep in subjects with EDS may limit the physiological drop in nocturnal blood pressure during sleep (nondipping pattern), favoring the subsequent occurrence of diurnal hypertension oxidative stress, endothelial dysfunction, systemic inflammation and subsequent CVD events. Interactions between hypertension and EDS for future CVD events were thus suspected, but our sensitivity analysis does not support this hypothesis. The presence of subclinical atherosclerosis may also contribute to our results; however the association between frequent EDS and CVD events was only slightly decreased after adjusting for the presence of carotid plaques.

### Study Limitations and Strengths

The present study has some limitations. Excluded subjects were older and with more chronic diseases. The prevalence of sleep complaints may be thus underestimated in our population with consequences on the associations between sleep disturbances (especially insomnia symptoms) and CVD events being finally potentially underestimated. Exposure variables including past-history of CVD were self-reported. Sleep complaints were only assessed once at baseline, excluding the possibility of assessing the evolution of sleep complaints over time and their association with CVD events. The presence of sleep apnea was not evaluated, thus the confounding effect of an underlying OSAS cannot be excluded, although our analyses were adjusted for snoring and BMI. Unfortunately, sleep duration and sleep deprivation were not available for this study and the report of sleep complaints in number of days affected per week was not possible. The Epworth sleepiness scale (ESS) which provides a measurement of the subject’s general level of daytime sleepiness [26] was used in one of the three study centers only (Montpellier). Of the Montpellier sample, only 27.8% of participants who declared having frequently or often EDS had an ESS score greater than 10. However, to our knowledge, the ESS scale and its pathological cut-off have never been validated in the elderly population specifically.

The present study also benefits from several strengths, notably sample size, adjustment for a large number of potential confounders including socio-demographic and lifestyle factors, depression and sleep medication. CVD events were defined according to standardized criteria and clinical verification sought from medical records. We were able to assess associations with multiple CVD end points including CHD *vs.* stroke, and fatal *vs.* non fatal events.

### Conclusions

In contrast to insomnia complaints, self-reported frequent EDS increased the risk for first and recurrent CVD events in the elderly. As EDS and insomnia complaints were largely reported in subjects with a past-history of CVD, we suggest that insomnia complaints are probably a consequence of CVD, whereas EDS is more likely to be a risk factor of CVD *per se*. With regard to the potential clinical utility of our observations, we would suggest that EDS may constitute a warning sign of increased risk of CVD in the elderly.
